# Factors influencing preclinical medical students’ satisfaction with hospital teachers’ instruction in a blended learning environment integrating the rain classroom platform in China

**DOI:** 10.3389/fpsyg.2025.1621120

**Published:** 2025-07-24

**Authors:** Dasheng Lu, Yuting Xiong, Liangjuan Yu, Yilin Bao, Xiaodong Teng, Yanyan Deng, Changping He, Hongxiang Zhang

**Affiliations:** ^1^Department of Cardiology, The Second Affiliated Hospital of Wannan Medical College, Wuhu, China; ^2^Vascular Diseases Research Center of Wannan Medical College, Wuhu, China; ^3^Outpatient Department, The Second Affiliated Hospital of Wannan Medical College, Wuhu, China; ^4^Human Resources Department, The Second Affiliated Hospital of Wannan Medical College, Wuhu, China; ^5^Anhui Medical University, Hefei, China; ^6^Department of Family Medicine, The Second Affiliated Hospital of Wannan Medical College, Wuhu, China

**Keywords:** rain classroom platform, blended learning, medical education, hospital teachers, student satisfaction, teaching evaluation

## Abstract

**Objective:**

The post-pandemic era has accelerated the integration of digital tools like the Rain Classroom platform into clinical medical education. This study examined factors influencing preclinical medical students’ satisfaction with hospital teachers’ instruction in this blended learning context at a Chinese medical college.

**Methods:**

A retrospective analysis of 278 teaching evaluations was conducted. Preclinical medical students anonymously assessed clinicians through an online teaching evaluation system across three domains: Professionalism (Score1, 30 points), Pedagogical skills (Score2, 40 points), and Learning outcomes (Score3, 30 points). Internal consistency was verified (Cronbach’s *α*: Score1 = 0.79, Score2 = 0.83, Score3 = 0.84, total score = 0.95).

**Results:**

We found that class size negatively correlated with all scores (Score1: rho = −0.186, *p* = 0.002; Score2: rho = −0.210, *p* < 0.001; Score3: rho = −0.225, *p* < 0.001). Specialized courses significantly increased odds of high Score3 (OR = 1.928, 95% CI [1.153–3.222], *p* = 0.012) compared to introductory courses. The results revealed that class size and course specialization were significant factors influencing students’ overall satisfaction, as indicated by their scores. Teacher demographics (age/gender/degree/title) showed no significant associations (all *p* > 0.05).

**Conclusion:**

Smaller class sizes and specialized courses enhanced satisfaction in Rain Classroom-based blended learning, while instructor characteristics like gender, age, degree, experience, and title did not influence students’ satisfaction.

## Introduction

Medical education encompassed both foundational disciplines and clinical coursework (Merlin et al., 2014; Albert et al., 2024), with the latter often requiring the active involvement of clinicians. However, clinicians and full-time faculty members in basic sciences may exhibit differences in teaching methodologies, formats, and pedagogical perspectives. Especially for doctors affiliated with university hospitals, teaching effectively grew tougher due to heavier clinical duties and less teaching time (Ramani and Leinster, 2008). Active learning strategies such as flipped classrooms (Marshall and Conroy, 2022) and team-based learning (De Vries et al., 2025) have been widely adopted in medical education to improve teaching effectiveness. During the COVID-19 pandemic, e-learning emerged as a prevalent educational modality, minimizing direct contact and ensuring continuity of education (Beheshti Fard et al., 2022; Ali et al., 2021; Khalil et al., 2020). Nowadays, blended learning, which combines traditional face-to-face teaching methods with e-learning, has become a staple in higher education (Mishra and Dholakia, 2023). This instructional blended learning model focused on fostering students’ autonomous learning, encouraging active participation, and promoting communication and collaboration (Yang et al., 2024; Jawaid et al., 2024). Research indicated that most medical students held favorable views on the benefits of blended learning (Shelly et al., 2024; Gong et al., 2021). The Rain Classroom mobile application, which was jointly developed with the Online Education Office of Tsinghua University in 2016, stood as one of the most extensively utilized e-learning tools (Lv et al., 2024). Students could swiftly access the Rain Classroom platform by inputting the lesson code shown on the projection screen or scanning the corresponding QR code via WeChat, China’s predominant social media application (Yang and Yu, 2022). After logging in by scanning the code, students can view the teacher’s teaching PPTs. The platform also supported pre-class preview, in-class interaction, and post-class review. Rain Classroom’s real-time quiz and bullet-screen discussion features facilitated interactive case-based learning, and the platform has been widely adopted by medical schools in China (Feng et al., 2022; Luo et al., 2024). The widespread use of smartphones and medical educational apps among preclinical medical students not only leveraged their ‘learn anywhere’ convenience but also facilitated peer interactions, communication, and collaborative learning. Despite these merits, the implementation of blended learning, particularly in clinical education where hospital-based teachers play a pivotal role, presents unique challenges and opportunities (Rowe et al., 2012). The differing teaching styles and clinical commitments of hospital teachers may significantly impact students’ learning experiences and, consequently, their satisfaction with the teaching process (Hashim et al., 2023). Understanding the factors that influence students’ evaluations or satisfaction with hospital teachers in a blended learning environment is crucial for optimizing clinical education.

Contemporary approaches emphasized that effective learning is situational (contextualized in clinical environments), continuous (supported by just-in-time mobile tools), and life-long (fostering self-directed skills). This study aimed to explore the factors that affect preclinical students’ satisfaction with hospital teachers’ instruction within a blended learning context integrating the Rain Classroom Platform. By analyzing teaching evaluation scores from a medical school affiliated with a hospital in China, we seek to identify key variables that contribute to student satisfaction.

## Methods

### Design

The current study involved third-year MBBS students and those required to take clinical medicine courses at Wannan Medical College. They were taught by clinicians from the Second Affiliated Hospital of Wannan Medical College for a minimum duration of one academic semester. The instructional approach adopted was a blended model, integrating traditional teaching methods with the Rain Classroom platform, aiming to enhance the learning experience and outcomes.

#### Participants (hospital teachers)

A total of 278 records were included in the analysis. The characteristics of teachers were shown in Table 1. The instructors ranged in age from 29 to 58 years, with an average age of 40.9 ± 6.7 years. Among them, 177 were male, accounting for 63.7% of the total. In terms of professional titles (technical title), there were 20 instructors with junior titles, 104 with intermediate titles, and 154 with senior titles, representing 7.2, 37.4, and 55.4% of the total, respectively. Regarding educational backgrounds, 147 instructors held bachelor’s degrees, 120 held master’s degrees, and 11 held doctoral degrees, accounting for 52.9, 43.2, and 4.0% of the total, respectively. The average teaching experience was 7.8 ± 3.9 years. Among the instructors, 146 (52.5%) were involved in specialized course instruction, while 132 (47.5%) taught non-specialized course, like introductory clinical medicine courses.

**Table 1:** Characteristics of the participating teachers.

Age (years)	40.9 ± 6.7		
Gender	Male	Female	
*N*	177	101	
Degree	Bachelor’s	Master’s	Doctoral
*N*	177	120	11
Teaching experiences	7.8 ± 3.9 years		
Professional titles	Junior	Intermediate	Senior
*N*	20	104	154
Courses	Specialized course	Non-specialized course	
*N*	146	132	

#### Participants (preclinical students)

The study analyzed teaching evaluation records at the class level, encompassing all third-year preclinical medical students who completed courses taught by hospital clinicians during the study period (September 2022–September 2024). Consequently, individual student demographic data (e.g., exact age or gender per evaluation) were not collected as part of the anonymized aggregate evaluation process. Third-year preclinical medical students in China typically range from 20 to 22 years old. Consistent with the general gender ratio observed across Chinese medical schools, the student cohort is estimated to comprise approximately 55% female and 45% male students.

### Procedures

A census sampling approach (total population sampling) was employed. All eligible class ratings were utilized for the analysis. Class sizes varied, with the smallest consisting of one small class and the largest comprising five small classes. Each small class generally has no more than 30 students, with most ranging between 25 and 30.

The evaluation instrument was administered through a dedicated online teaching evaluation system, where students could voluntarily participate in the online evaluation of teaching. Only the administrative staff of the Education Department can access the rating data, while ordinary teachers or students are unable to obtain it.

#### Questionnaire structure

Section 1 (Score1): 5 items on teacher professionalism (e.g., punctuality, content mastery).

Section 2 (Score2): 5 items on pedagogical skills (e.g., interaction clarity, technology use).

Section 3 (Score3): 3 items on learning outcomes (e.g., concept mastery, interest stimulation).

The evaluation instrument was developed by a panel of medical education experts and was contextualized for blended learning environments using Rain Classroom. The Scoring Criteria was shown in Supplementary Table 1. The reliability of the three core components (Score1, Score2, and Score3) within the electronic evaluation system was assessed using Cronbach’s alpha (*α*), a measure of internal consistency (Shrestha, 2021; Zhao et al., 2024). Cronbach’s α > 0.70 value falls under acceptable limits whereas >0.80 would be classified as a good reliability score. The total score for each evaluation was 100 points, with each of the three components contributing 30, 40, and 30%, respectively. To further investigate, participants were categorized into a high-score group, comprising those with scores above the average, and a control group, encompassing all other participants.

### Grouping

The main courses were divided into two parts: one part consists of specialized courses such as internal medicine, surgery, gynecology, pediatrics, etc., and the other part non-specialized courses, like introduction to clinical medicine or an overview of clinical diseases. These courses were taught by clinical specialists in their respective fields, ensuring the relevance and practicality of the instruction.

To gain a deeper understanding of the factors influencing student evaluations, we included various teacher attributes and course characteristics in our analysis. These factors included the teachers’ age, gender, academic degree, professional title, teaching experience (in years), class size, and the specific course being taught.

### Statistical analysis

Cronbach’s alpha values for internal reliability was displayed in Supplementary Table 2 (all exceeded 0.70). We employed both descriptive and inferential statistics to analyze the data.

#### Descriptive statistics

We calculated means and standard deviations for continuous variables (e.g., teachers’ age, teaching experience, students’ evaluation scores), constructed frequency distributions for categorical variables (e.g., teachers’ gender, academic degree, professional title, course type) and summarized descriptive statistics for the three core components of student evaluations (Score1, Score2, and Score3).

#### Inferential statistics

We conducted independent samples t-tests to compare means between groups (e.g., high-score group vs. control group), applied chi-square tests to assess associations between categorical variables and performed Spearman correlation analysis to examine relationships between continuous variables.

We also utilized logistic regression to identify factors influencing high scores in student evaluations, controlling for potential confounding variables. Statistical significance was defined as a *p*-value less than 0.05. The analysis was conducted using SPSS 22.0 software.

### Ethical approval

This retrospective analysis has been approved by the institutional academic ethics committee of The Second Affiliated Hospital of Wannan Medical College, and as it does not involve human intervention or information disclosure, informed consent is not required. The study was designed and conducted in accordance with the principles of the Declaration of Helsinki.

## Results

### Students’ teaching evaluations and the influencing factors

The average participation rate in the students’ evaluations was 50.73%. Overall, the average scores given by students to their teachers were relatively high, with a minimum score of 86.5 and a maximum score of 100. We divided the scores into a High-Scoring Group (HS group, above the average score) and a Control Group. As shown in the Table 2, there were no significant differences in the average age (41.5 ± 6.7 vs. 40.6 ± 6.6, *p* = 0.287), gender composition (male: 62.3% vs. 64.7, *p* = 0.674), degree distribution (*p* = 0.492) or teaching experience (7.8 ± 3.9 vs. 7.9 ± 4.0, *p* = 0.907) between the two groups (*p* > 0.05). However, significant differences were observed in terms of courses (*p* = 0.007), class size (*p* < 0.001), and professional titles (*p* = 0.004). The HS group had a higher proportion of junior titles, a greater proportion of smaller class sizes, and a relatively higher proportion of specialized courses.

**Table 2:** Comparison of teachers’ attributes between the high-score group and control group.

	Control group	High score group	*p* value
Number	122	156	NA
Age (years)	41.5 ± 6.7	40.6 ± 6.6	0.287
Male (*n*, %)	76 (62.3%)	101 (64.7%)	0.674
Professional titles (junior/intermediate/senior)	2/52/68	18/52/86	0.004
Degree (bachelor’s/master’s/doctoral)	67/52/3	80/68/8	0.492
Class size (1/2/3/4/5)	7/2/38/69/6	32/36/0/73/15	<0.001
Course (non-specialized course/specialized courses)	69/53	63/93	0.007
Teaching experience (years)	7.8 ± 3.9	7.9 ± 4.0	0.907

**Figure 1:**
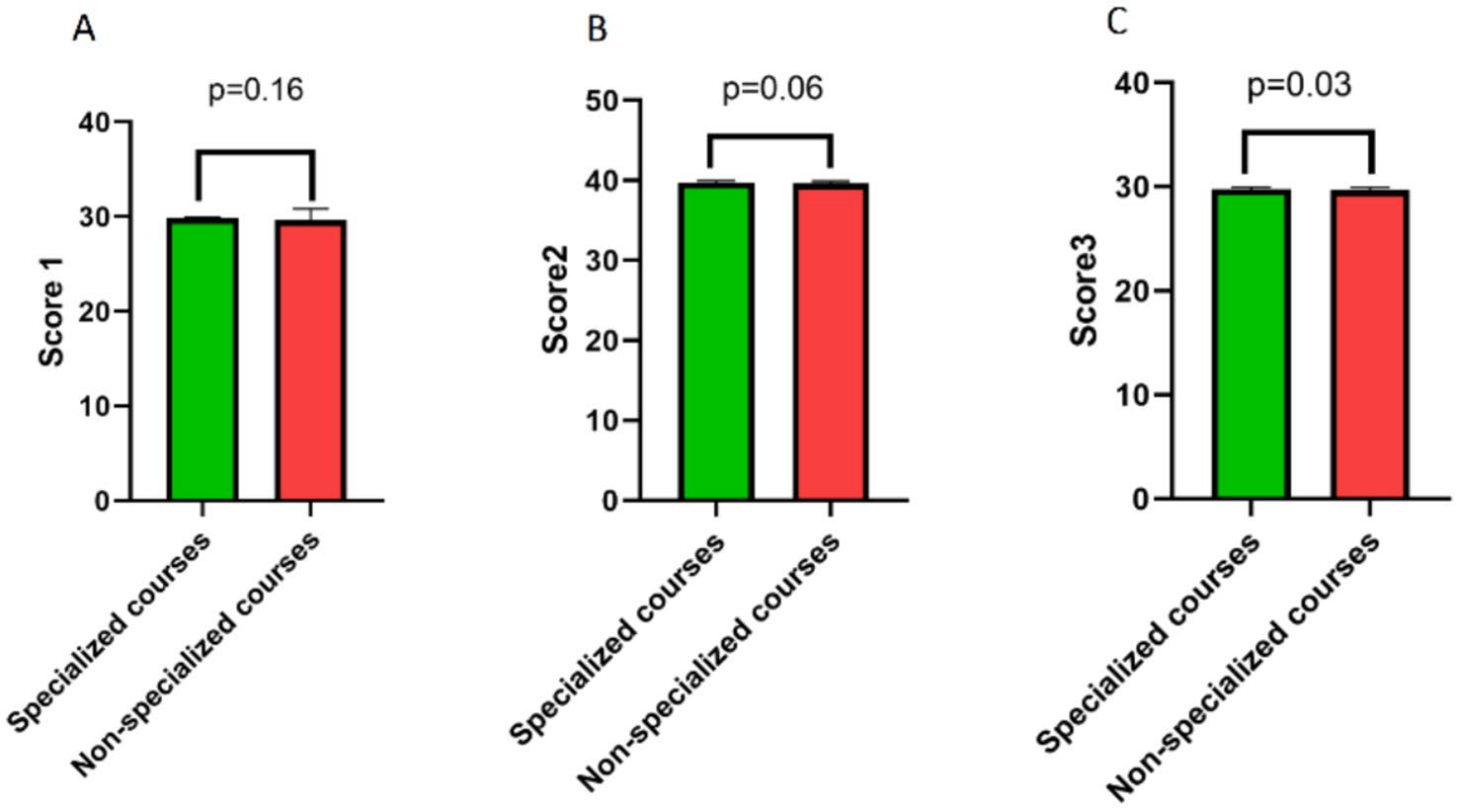
Differences in various categories of student assessment scores between course specialization group and the non-specialization group. There was no significant difference in Score1 between the course specialization group and the non-specialization group **(A)**, nor was there any significant difference in Score2 **(B)**. However, the Score3 of the course specialization group was higher than that of the non-specialization group **(C)**.

In a multivariate analysis model without adjusting for other factors, class size (OR: 0.745, 95%CI: 0.592 ~ 0.937, *p* = 0.012) and course specialization (OR: 1.776, 95%CI: 1.079 ~ 2.924, *p* = 0.024) emerged as the primary factors influencing high scores (Table 3). In a multivariate analysis adjusted for gender, age, teaching experience, and degree, class size (OR: 0.752, 95%CI: 0.594 ~ 0.951, *p* = 0.017) and course specialization (OR: 1.928, 95%CI: 1.153 ~ 3.222, *p* = 0.012) remained independent factors affecting the likelihood of achieving high scores (Table 3). After identifying the impact of class size and course specialization on students’ overall scores, we further explored how they specifically affect individual score components. Correlation analysis revealed that class size was negatively correlated with Score1 (rho = −0.186, *p* = 0.002), Score2 (rho = −0.210, *p* < 0.001), and Score3 (rho = −0.225, *p* < 0.001) (Table 4). In contrast, compared to the non-specialization group, the course specialization group showed a significant increase only in Score3 (*p* = 0.03) (Figure 1).

**Table 3:** Multivariate analysis of factors influencing teaching evaluation scores.

Model1				
	B	Exp(B)	*p*	95% CI
Professional titles*			0.145	
Professional titles (intermediate)	−1.494	0.225	0.065	0.046 ~ 1.099
Professional titles (senior)	−1.229	0.293	0.126	0.061 ~ 1.414
Course specialization	0.574	1.776	0.024	1.079 ~ 2.924
Class size	−0.295	0.745	0.012	0.592 ~ 0.937
Constant	2.196	8.988	0.007	

Model2				
	B	Exp(B)	*p*	
Professional titles*			0.124	
Professional titles (intermediate)	−1.625	0.197	0.051	0.039 ~ 1.004
Professional titles (senior)	−1.413	0.244	0.107	0.044 ~ 1,357
Course specialization	0.656	1.928	0.012	1.153 ~ 3.222
Class size	−0.285	0.752	0.017	0.594 ~ 0.951
Constant	1.985	7.279	0.077	

Based on the results of the univariate analysis, statistically significant indicators were included in the multivariate analysis. Model1 was not adjusted for other factors, while Model2 was adjusted for gender, age, teaching experience, and degree. *Professional titles are set as dummy variables, with junior title as the reference.

**Table 4:** Result of Spearman’s rho between class size and different types of student evaluation scores.

	Spearman’s rho	*p* value
Score1	−0.186	0.002
Score2	−0.210	<0.001
Score3	−0.225	<0.001

## Discussion

This study found that in medical education, utilizing hospital doctors as instructors to teach senior students through a blended learning mode combining traditional teaching with e-learning (i.e., Rain Classroom) achieved high student evaluations or satisfaction. The evaluations covered three core areas: Professionalism (Cronbach’s *α*: 0.79), Pedagogical Skills (Cronbach’s α: 0.83), and Learning Outcomes (Cronbach’s α: 0.84). When scores 1, 2, and 3 were combined into a total score, the Cronbach’s alpha coefficient reaches 0.95, demonstrating excellent internal consistency. Comparatively, the size of the class and the subject matter (content) of the course influenced student ratings or learning outcomes, whereas factors such as the instructor’s gender, age, degree, teaching experience, and professional title did not appear to have a significant independent impact on student ratings.

Student evaluation of teaching has been a widespread practice in higher education institutions for a long time. While its popularity has been on the rise, student evaluation of teaching is also regarded as a crucial yet contentious instrument for improving teaching quality (Spooren and Mortelmans, 2006). In many universities, it has become obligatory to utilize student evaluation of teaching for faculty assessment, as well as for making personnel decisions regarding retention, promotion, and also for ensuring quality assurance and accreditation. Studies have investigated whether various teacher characteristics, such as age and gender, influence student evaluation of teaching. Regarding the age factor, research has yielded two differing outcomes. Wilson et al. reported that ratings of teaching effectiveness diminished with increasing age (Wilson et al., 2014), while Shah et al. discovered that there was no statistically significant preference among students regarding the age of their teachers (Shah and Udgaonkar, 2018). Concerning the gender factor, studies conducted so far have also produced varying results. Mengel et al. analyzed a quasi-experimental dataset comprising 19,952 student evaluations, revealed that female teachers received lower evaluations compared to their male counterparts (Mengel et al., 2019). However, Daud et al. found no significant disparities in rated teaching effectiveness based on the instructor’s gender (Nl, 2011). Research conducted thus far has yielded conflicting results regarding the correlations between age, gender, and perceived teaching effectiveness, suggesting that the influence of age and gender on student evaluation of teaching varies across different populations.

In the present study, in medical education where blending teaching is adopted as the instructional mode and clinicians serve as instructors, we have found that the age and gender of the teachers do not impact student evaluations. This suggests that these students do not seem to regard the age and gender of the hospital teachers as significant factors in determining teaching effectiveness. We also found that the highest degree of the teacher has no significant impact on student evaluation scores, which is similar to previous research findings (Pama et al., 2013).

Previous findings revealed that the correlation between the total number of years of teaching experience and teacher effectiveness, when assessed by improvements in student achievement, was intricate, subtle, and non-linear (Irvine, 2019). In our analysis, we found no significant correlation between teachers’ teaching experience and students’ ratings, indicating that in the blending teaching of clinical medicine courses, which are relatively new, experienced teachers did not necessarily hold a considerable edge. Another aspect reflecting a clinician’s qualifications is their technical title. We found that although in the univariate analysis, the group scoring above average had a higher proportion of junior titles, in the multivariate analysis, technical title was not an independent factor influencing the score grouping. This suggests that technical title does not significantly affect teaching effectiveness or student ratings in medical education.

Compared to the aforementioned indicators that showed no significant differences, our analysis revealed that class size was a factor that influences student teaching ratings, both in univariate and multivariate analyses. This suggests that the impact of class size on teaching effectiveness needs to be considered in blending teaching for clinical courses. As medical curricula are extensive, third-year students are exposed to specialized courses such as Internal Medicine, Surgery, Gynecology, and Pediatrics, as well as introductory courses like Introduction to Clinical Medicine. Our analysis found that teachers received higher scores in specialized clinical courses compared to the Introduction to Clinical Medicine course. The reasons for this are not yet clear. It could be that both teachers and students may not attach enough importance to non-specialized courses like Introduction to Clinical Medicine, or it could be that non-specialized courses cover a wide range of topics and require relatively more class hours to adequately cover the material. After examining the grading criteria, we discovered that the only area where there was a notable discrepancy was in Score3, which assesses students’ understanding and mastery of the core concepts covered in the course, along with the teacher’s ability to ignite students’ interest and passion for learning. As a result, we hypothesize that teachers may need to dedicate more energy or instructional hours to non-major courses.

### Limitations

Firstly, it should be noted that this analysis is based on teaching data from just one medical college in China, and thus the findings may not necessarily apply to the whole country. Secondly, while our study did not identify age, gender, degree, and title as independent factors influencing student evaluations, it is possible that interactions among these factors could play a role in shaping teaching ratings. Furthermore, there may be other teacher attributes that were not considered in our analysis, so the results should be interpreted with caution. Thirdly, student teaching ratings can be subject to various influences, including the quality of the survey instrument itself, and may not always accurately reflect teaching effectiveness or quality. It is worth noting that the study findings could also have been influenced by course design and e-learning platform used, students’ perception of usefulness of the blended learning approach along with their cognitive presence in the online environment. Besides, the lack of pre-intervention student assessments and teacher feedback limits contextual interpretation. Future studies should integrate baseline surveys and teacher interviews to triangulate findings.

## Conclusion

Smaller class sizes and specialized courses enhanced satisfaction in Rain Classroom-based blended learning, while instructor characteristics like gender, age, degree, experience, and title did not influence students’ satisfaction. In blended teaching, small-class teaching and specialized course instruction should be implemented more extensively.

## Data Availability

The raw data supporting the conclusions of this article will be made available by the authors, without undue reservation.
