# Supplementary material for: Factors influencing preclinical medical students’ satisfaction with hospital teachers’ instruction in a blended learning environment integrating the rain classroom platform in China
Source: Front Psychol. 2025 Jul 24;16:1621120. doi: 10.3389/fpsyg.2025.1621120 (PMC12315698; doi:10.3389/fpsyg.2025.1621120)
Supplement: Supplementary file 1 [file Table_1.docx]

**Supplementary Table 1. Scoring Criteria for the Teaching Evaluation Instrument**

| **Domain** | **Max Points** | **Assessment Criteria** |
| --- | --- | --- |
| **Professionalism (Score1)** | 30 | • Follows national education policies and role models • Shows serious teaching attitude and preparation • Masters course content thoroughly  •Maintains punctual class schedules  •Avoids arbitrary teaching changes |
| **Pedagogical Skills (Score2)** | 40 | • Integrates ideological education into teaching • Teaches professional ethics and life principles • Uses flexible teaching methods and technologies  •Delivers logically structured explanations  •Facilitates interactions and after-class support |
| **Learning Outcomes (Score3)** | 30 | • Mastery of core knowledge points • Stimulated learning interest/passion • Overall teaching satisfaction |
| **Total Score** | 100 | •Score1  •Score2  •Score3 |

The scoring system can be accessed via the website: <https://wnyxyedu.mh.chaoxing.com/>
