# Supplementary material for: Factors influencing preclinical medical students’ satisfaction with hospital teachers’ instruction in a blended learning environment integrating the rain classroom platform in China
Source: Front Psychol. 2025 Jul 24;16:1621120. doi: 10.3389/fpsyg.2025.1621120 (PMC12315698; doi:10.3389/fpsyg.2025.1621120)
Supplement: Supplementary file 2 [file Table_2.docx]

| **Scale** | **Cronbach's α** | **Interpretation** | **No. of Items** |
| --- | --- | --- | --- |
| **Score1** | 0.79 | Acceptable | 5 |
| **Score2** | 0.83 | Good | 5 |
| **Score3** | 0.84 | Good | 3 |
| **Total score** | 0.95 | Good | 3 |

**Supplementary Table 2. Reliability Analysis of Evaluation Scales**

α > 0.70:

Acceptable

α > 0.80: Good

Data was calculated based on a sample of 30 students (validation).
